# The meteorology of cytokine storms, and the clinical usefulness of this knowledge

**DOI:** 10.1007/s00281-017-0628-y

**Published:** 2017-04-27

**Authors:** Ian A. Clark, Bryce Vissel

**Affiliations:** 1 Research School of Biology, Australian National University, Canberra, Australia; 2 School of Life Sciences, Faculty of Science, University of Technology, Sydney, Australia; 3 Garvan Institute of Medical Research, Sydney, Australia

**Keywords:** Cytokine storm, TNF, Il-1, Physiology, Innate immunity, Disease pathogenesis, Neurodegenerative diseases

## Abstract

The term cytokine storm has become a popular descriptor of the dramatic harmful consequences of the rapid release of polypeptide mediators, or cytokines, that generate inflammatory responses. This occurs throughout the body in both non-infectious and infectious disease states, including the central nervous system. In infectious disease it has become a useful concept through which to appreciate that most infectious disease is not caused directly by a pathogen, but by an overexuberant innate immune response by the host to its presence. It is less widely known that in addition to these roles in disease pathogenesis these same cytokines are also the basis of innate immunity, and in lower concentrations have many essential physiological roles. Here we update this field, including what can be learned through the history of how these interlinking three aspects of biology and disease came to be appreciated. We argue that understanding cytokine storms in their various degrees of acuteness, severity and persistence is essential in order to grasp the pathophysiology of many diseases, and thus the basis of newer therapeutic approaches to treating them. This particularly applies to the neurodegenerative diseases.

## Introduction

As previously discussed [1, 2], the term cytokine storm appears to have been first used to describe the chaotic pathophysiological state encountered during an acute graft-versus-host disease [3]. The term has caught the popular imagination, with >160,000 hits on a search engine. A flood of harmful polypeptides bursting forth from our cells making us very ill, as if we had severe influenza, is a powerful and accurate image, one clearly worth understanding. Over the intervening 24 years the activity of these cytokines has encompassed a much wider array of biology than dreamed of in 1993, and it is now timely to step back and examine the concept again. In order to provide a wider understanding of the phenomenon of a cytokine storm, we have recounted the history of researchers’ awareness of the main mediators whose release initiates the process. Although the popular concept of a cytokine storm is about dramatic illness and disease, these mediators also have essential roles in normal physiology and innate immunity. The principles are the same throughout, but site of production, cytokine concentration and persistence, as well as generation of countering cytokines, determine outcome. The perspectives now possible thus allow us to develop a better understanding of both of these aspects in health and in sickness, as well as focus on the therapeutic possibilities of this knowledge, which is a main theme of this text. We also extend the concept of a cytokine storm into the chronic neurodegenerative states. These diseases remind us that unrelenting moderate rain can destroy, just as surely as a massive storm that causes a flash flood.

## Cytokines, the raw material of a cytokine storm

Some 50 years ago the idea of unsuspected secreted polypeptides, mediators akin to hormones but arising throughout the body from cells with other primary functions rather than from specialized secretory organs, and communicating with many other cells, was quite new. The word cytokine became a generic term for these molecules once immunologists realized that classifying them according to their cell of origin, such as lymphocytes releasing lymphokines [4] and monocytes releasing monokines [5] was quite inadequate, particularly since a single mediator could have many cellular origins, as well as pleotropic activity. By 1991, for example, tumor necrosis factor (TNF) had been documented to possess, or at least initiate, 50 biological functions, most demonstrated quite soon after it had been cloned and made available in a recombinant form [6]. TNF [7] is one of the cytokines that kept its original function-based name. Others include interferons (IFNs) [8] – IFN-γ [9] being particularly relevant – lymphotoxins (LTs) [10] and the transforming growth factors (TGFs) [11]. We should acknowledge here the insight of the authors of a 1979 Letter-to-the-Editor in the Journal of Immunology [12] who proposed the interleukin (IL) form of nomenclature as a way to rescue order out of chaos by suggesting that a number of acronyms describing observed activities could be grouped under a numerical system, beginning with IL-1 and IL-2. This suggestion proved its worth with the increased molecular definition of entities previously known only as activities. Currently IL-1 to IL-37 are recognized, and most, if investigated, are likely to be released in a severe cytokine storm. From what is known of their proinflammatory links to TNF, the cytokines IL-1, IL-12 [13] and IL-17 [14] are particularly likely to contribute to the dramatic outcome of a full-blown storm, but this is the topic of a much wider ranging review. This text is largely restricted to TNF and IL-1, cytokines that conspicuously are released early, and readily induce others. A broad canvas of the array of mediators that can, in excess, form a cytokine storm can be impressive, as can the mathematical models of its dynamics [15], but their known interactions are still in a state of flux. In order to tell the story of the storm rather than the raindrops, we focus here on studies that have contributed to allowing the master initiators, which are released early and set the process going, to be understood, and thus become therapeutic targets.

## The biological antiquity of two main players, TNF and IL-1

When a molecule present in mammals proves also to be found in earlier, particularly very early, life forms it is generally taken to denote that it has been conserved in the biological repertoire because it is essential to life. Cytokines can be good examples. For instance, although TNF is large in the mammalian literature it is also found in the marine sponge, *Chondrosia reniformis* [16], and the reef-building corals, *Acropora* spp. [17]. Remarkably, these authors found that coral TNF has a mutual receptor cross-reactivity with human TNF. Similarly, IL-1 is present in the starfish, *Asterias forbesi* [18]. Among insects, cells from at least two genera of moths contain IL-1 and TNF [19], so conceivably all Lepidoptera are similarly endowed. Lower vertebrates such as fish also generate both of these cytokines [20, 21], as well others such as IL-6 [22], IL-8 [23], IL-12 [24] and IL-17 [20].

## The discovery of TNF and IL-1 arose from investigating endotoxicity

The earliest published record relevant to this story seems to have been the observation by Maegraith in his 1948 monograph [25] that the functional effects of parenteral bacterial endotoxin, in the form of the typhoid vaccine of the day [26], can be equated with the range of clinical changes observed in falciparum malaria. The clinical non-specificity of malaria, which can mimic rickettsial, bacterial, viral, and non-infectious diseases, carrying with it implications of equal non-specificity of disease mechanism, has been documented for many decades [27]. Predictably, therefore, whatever mediates the complex illness seen in endotoxicity is acting in these other diseases as well. This conceptual framework has persisted, and continues to be strengthened. The circumstances to which it has now been extended go far beyond its conceptual origins, where gram-negative bacteria, the walls of which contain endotoxin, are present. A crucial next advance was the argument, in 1957, that the pathophysiology of endotoxicity, traumatic shock and hemorrhagic shock are remarkably identical, even to the point of experimentally inducing tolerance to any one of these states protecting against all three [28, 29]. The first arises in bacterial infection, but the other two are decidedly non-infectious.

Although cytokines now loom large in immunology, awareness of them emerged from curiosity about the useful and harmful (endotoxic) effects of bacterial endotoxin. Originally found, as noted above, in cell walls of gram-negative bacteria, endotoxin proved to be a form of lipopolysaccharide (LPS). The term was used interchangeably with LPS for many years, the later taking precedence in recent times. Endotoxin/LPS influences larger life forms in what can seem a bewildering number of ways. Intriguing early phenomena included the in vivo tolerance to its effects observed after a second and subsequent injections [30]. Another major development in 1955 was the capacity of typhoid vaccine, inevitably, we now know, containing LPS, to generate a mediator termed endogenous pyrogen in the serum of rabbits [31]. This was argued to be of leukocyte origin, to be transferable to other rabbits, and not to cross-tolerize with typhoid vaccine [32]. In due course this was one of the paths that led to the recognition of IL-1, as discussed below.

Further complexity arose from the long history of experimental anti-tumor activity [33] of bacterial LPS implying a biological relevance beyond bacterial disease. Others [34] had achieved similar outcomes with an attenuated form of *Mycobacterium bovis* termed Bacillus Calmette-Guérin (BCG), which greatly sensitizes mice to LPS [35]. Combining these anti-tumor approaches eventually led, in 1975 [7], to the term tumor necrosis factor (TNF), currently with over 135,000 PubMed hits, far more than any other cytokine, entering the scientific lexicon. Until the insights that arose from this work, endotoxin/LPS had been assumed to be directly responsible for its tumor-killing activities. The next year the hypoglycemia induced by LPS [36] proved to arise, as did TNF, through the actions of a macrophage-origin, LPS-induced, polypeptide the authors termed glucocorticoid antagonizing factor (GAF) [37]. As far as its purification was taken, GAF mirrored TNF [38]. These two lines of research pointed the way to appreciating that endotoxin, of itself, is not, as had been thought, directly active against tumors or has the ability to change blood glucose levels, but induces soluble mediators of host origin that have these properties. In other words, mice exposed to parenteral endotoxin generate molecules with anti-tumor and hypoglycemic activity. This opened minds to wondering how many of these mediators existed, and what they did.

Work on the identity of endogenous pyrogen (see [31], above) lay dormant for many years, until 1979, when LPS was appreciated to induce serum amyloid A (SAA) from macrophages indirectly, through the action of another macrophage-origin mediator the authors appropriately termed SAA-inducer [39]. Soon after, this product was found to be identical to both endogenous pyrogen and lymphocyte activating factor (LAF) [40, 41], one of the many names subsumed into the term IL-1 [12]. Hence endogenous pyrogen was evidently identical to IL-1.

## What controls TNF increases in innate immunity and disease?

As we have recently reviewed in this context [42], from the late 1980s interest in the evolution of the immune system fostered ideas on how the cytokines that caused innate immunity and disease pathogenesis could arise, and be controlled, in non-infectious as well as infectious disease states. The models that this era generated [43, 44] are still in place. In brief, pathogen-associated molecular patterns (PAMPs) are released by pathogens, and damage-associated molecular patterns (DAMPs) by tissue damaged by trauma, hypoxia, and metals such as lead, which hypomethylate host DNA. This has the same PAMP activity as do mitochondrial DNA and bacterial DNA, which are innately hypomethylated. Both PAMPs and DAMPs are agonists for non-specific pattern recognition receptors (PPRs) on or inside most cell types, the best described of which are the toll-like receptors, or TLRs [45]. In this way, a disparate collection of signals triggering the same functional outcome fits within a framework that provides these signals with the ability to trigger the release of proinflammatory cytokines. For instance, within this now widespread terminology the LPS discussed in the previous section is one of the PAMPs that are agonists for TLR4 [46]. These cytokines, through the processes of innate immunity, have the capacity to kill the pathogen that provided the PAMP. Also, when in excess, these cytokines initiate pathological processes shared by both infectious and non-infectious diseases.

## Some physiological roles of TNF and IL-1 outside the brain

While the principles governing cytokines in innate immunity and disease pathogenesis were being elucidated it was not yet appreciated that TNF and IL-1 are crucial for physiological homeostasis [47]. IL-1 is generally less reported in this context than is TNF, arguably because IL-1-specific reagents are less freely available. Nevertheless, normal hematopoiesis is known to depend on these two cytokines [48, 49], as does normal sleep regulation [50]. Mitochondrial function depends on TNF [51], and it has, as we have reviewed, homeostatic effects on the normal reproduction rate of various progenitor cells, of particular clinical importance those of endothelial cells in severe malaria and sepsis [52]. Clearly, much physiology is controlled by small fluctuations in TNF and IL-1 acting as signaling molecules. The above examples suffice to demonstrate the now established principle that the key cytokines mediating innate immunity and disease pathogenesis are, as next discussed, normally present, and indeed necessarily present, in healthy individuals, fulfilling physiological roles unrelated to inflammation. Thus we advocate not routinely referring to TNF and IL-1 as proinflammatory cytokines, since this terminology often leads this closely linked pair to be regarded simply as biomarkers for the presence of inflammation, a link not made until over 10 years after TNF had been first described [53], and in any event relatively minor within the range of broad biological effects of these cytokines.

## Roles of TNF and IL-1 in innate immunity and disease pathogenesis

As has been reviewed [54], in the mid-1970s our laboratory had been seeking a plausible explanation for our observation that mouse hemoprotozoan parasites die inside circulating erythrocytes during the immune response [55], an unpopular finding in certain quarters, since it was inconsistent with antibody-based dogma on which malaria vaccine research was then based. We were also wondering why a two week prior infection of mice with BCG, administered on the advice of a colleague, Jean-Louis Virelizier, created a milieu inimical to these parasites, causing their death, in the same intra-erythrocytic location, some 12 h after their injection into the mice [56]. An additional puzzle was why the illness and pathology caused by these organisms, was, as Maegraith had argued in human malaria decades earlier [25], so similar to endotoxicity. Importantly, we had found we could mimic the mouse disease as well as the death of parasites inside erythrocytes by injecting small amounts of endotoxin/LPS [57]. Remarkably, these changes happened of their own accord in undisturbed self-limiting infections, arguably induced by a stimulus functionally similar to LPS that was provided by the increased parasite load as the infection progressed. Thus we began to search for explanations based on LPS-induced macrophage products that might make sense of these observations. The essential revelation that awakened us to the possibility of TNF being responsible came from reading the then new1975 Sloan Kettering paper [7] on the use of BCG and LPS, reagents we had been using with the same timing, but in our case to understand the relationship between an infectious agent and its host rather than tumor killing. With their collaboration in assaying TNF, we began to develop our then novel view that infectious disease is caused not directly by the invading pathogen, but by what were effectively the side effects of the host’s over-exuberant innate immune response to it. We had evidence, albeit now regarded as rudimentary because of the pre-recombinant assays of the day, that TNF and lymphocyte activating factor (LAF), an old name for IL-1, could be involved [58].

This proposal could not, of course, be properly tested until peptides such as cytokines became available in recombinant form, and in sufficient quantity for in vivo use. Since TNF was first recognized for its tumor killing properties, ambitions were high in the late 1980s that rTNF [59] would be therapeutically valuable to cancer patients. In hindsight, we know that severe side-effects [60–62] of the type now attributable to a cytokine storm, were inevitable. To these researchers’ minds the side effects of rTNF they produced resembled an influenza-like disease [63], but to us, with our malaria background, they were also profoundly malaria-like. This generally unanticipated outcome of treatment with rTNF was extremely instructive for our theories on the cytokine origins of the illness of malarial disease and bacterial sepsis [58, 64], and we and others had already been injecting rTNF into animal models for this purpose [65, 66]. In the next year, 1988, these principles were incorporated into the original description of sickness behavior [67], which we subsequently described, in its extreme form, as being the basis of the syndrome seen in severe systemic protozoal, bacterial, viral disease, as well as post-trauma [68].

Thus developed the view that infectious disease is caused not directly by the invading pathogen, but by the host’s excessive innate immunity to it. The concept eventually spread across the board to include both innate immunity and disease pathogenesis in, for example, infection with *Mycobacterium* spp. [69, 70], *Brucella abortus* [71, 72], *Salmonella* spp. [73, 74], *Listeria monocytogenes* [75, 76], *Leishmania* spp. [77, 78], *Toxoplasma gondii* [79, 80], and influenza [81, 82]. This pattern of cytokines being useful in low concentrations but harmful in high, first reviewed in 1987 in a malaria context [83], is now generally accepted in infectious disease. It is not, however, at all restricted to pathogen-induced conditions, as discussed below.

Many reports link high circulating levels of TNF and IL-1, arguably the original hallmarks of a cytokine storm, with the biochemical details of the pathogenesis of clinical markers of critically ill patients. A random illustration is hypoalbuminemia, an independent marker of poor outcomes in severely ill patients with various diagnoses [84], as recently investigated in critically ill children in intensive care [85]. As reviewed [68], hypoalbuminemia is also a characteristic of malaria, sepsis, acute viral diseases, and severe trauma, all conditions with high TNF. They are, indeed, prototype examples of the effects of a cytokine storm. Reduced albumin is to be expected in all these circumstances, since the liver-specific albumin gene is positively regulated by Dbp [86], one of the circadian genes that TNF and IL-1 suppress [87]. Picomolar concentrations of TNF have been demonstrated to reduce albumin production by human hepatocytes [88]. Whether it does so by inducing IL-1, or independently, or whether they act synergistically, appears, as in too many other circumstances, yet to be determined.

## Persistent cytokine storms in the ill brain

Moderate, but persistent, cytokine storms are typical of the chronic neurodegenerative states, including post-stroke, post-traumatic brain injury, and Alzheimer’s disease (AD). In the weather analogy, cytokines are what water is to life. Light falls of rain, like low levels of TNF and IL-1, keep physiology ticking over and organisms alive. In moderate amounts rain improves outcome, as does self-limiting innate immunity, but in acute excess, or unrelenting moderate amounts, both rain and cytokines can kill. Both the acute and unrelenting patterns are valid cytokine storms. Acute systemic cytokine excesses, ie those outside the central nervous system (CNS), typically arise from the effects from bacterial or viral PAMPs, and are, if not acutely fatal, generally transient. In contrast, when excess cytokine is generated within the brain in sufficient quantities, as distinct from entering from outside, the usual type of cytokine storm is an unrelenting moderately raised activity that leads to non-resolving inflammation.

This non-resolving pattern of inflammation in the brain is consistent with the consequences of injecting LPS, the prototype TNF inducer, and a TLR4 agonist, in order to generate models of neurodegenerative disease in rodents [89]. Following a single systemic LPS injection, TNF production in mouse brain remains high for at least ten months [90]. In contrast, serum TNF levels peaked at the expected nine hours. Why this difference? These authors argue that an acute systemic injection of LPS activates brain microglia through TNF receptors that initiate sustained activation of brain cytokine synthesis and neuroinflammation. These studies are consistent with earlier work in which the TNF switch-off that occurs systemically after a second LPS injection, which demonstrates the presence of LPS tolerance, proved to be absent in the intracisternal space [91]. Evidently TNF generation is inhibited differently systemically and cerebrally, conceivably mediated either through failure of the anti-inflammatory cytokines IL-4 or IL-10 to increase as they do systemically, and, as noted above, LPS tolerance being weak or absent inside the blood brain barrier (BBB). It is also consistent with the activated state of microglia many years after brain ischemia [92] or brain trauma events [93, 94], as well as with evidence for a positive feedback loop for microglial activation via TNF [95]. Thus the central nervous system is especially vulnerable to cytokine storms that arise when TNF is generated within the brain, from many cell types, particularly microglia and astrocytes but also including neurons [96], and leads to loss of homeostasis in such vulnerable sites as synapses. One predictable consequence is the loss of the subtle homeostasis we depend on for learning, memory, and normal behavior, as seen in chronic neurodegenerative states. As discussed below, severe changes include neuronal death through excitotoxicity.

Since brain function determines subtleties such as personality, behavior, executive function, mood, willpower, learning and memory, we can expect the effects of brain TNF excess to be much more nuanced than the same change in the rest of the body. This is indeed what happens. For example, mice without certain TNF receptors do not become aggressive [97]. The origins of delirium, in which a seriously ill patient shows transient disorientation, confusion and memory loss as part of an exaggerated sickness behavior, remain controversial [98], but it is certainly part of a cytokine storm. It is now considered to be best understood in terms of peripheral TNF being increased sufficiently for enough to cross the BBB for a limited period [99]. Dementia, in contrast, reflects continual TNF production within the brain. Likewise, the coma that is often part of an encephalopathy accompanying sepsis, influenza or malaria can also be rationalized in cytokine terms, with associated coma argued to arise through increased cerebral TNF reducing orexin levels [100]. See reference [101] for a review.

## Some key physiological roles of TNF and IL-1 inside the brain

As we have noted [42], physiological roles of TNF and IL-1 inside the brain include their release during physiological neuronal activity and, as has been reviewed [102], playing a crucial role in regulating the strength of normal synaptic transmission. TNF, of itself rather than through the inflammatory cascade it can trigger, is also involved in normal transmission via modulating excitatory neurotransmission [103], trafficking of AMPA receptors [104], homeostatic synaptic scaling [105], long-term potentiation [106], and maintaining normal background levels of neurogenesis [107]. As noted earlier, and of particular relevance in the brain, which requires much oxygen, mitochondrial function depends on TNF [51]. So too does regulation of the neurotransmitter orexin [100], which, as we recently reviewed in a brain disease context [101], controls sleep, motor control, focused mental effort, appetite and water intake. TNF also regulates neuronal type-1 inositol trisphosphate receptors (IP3R), which are central to neuronal Ca^++^ homeostasis, and thus the ionic signaling cascades on which normal function of these cells depends [108]. Likewise, glycine receptors, which are structurally related to γ-aminobutyric acid (GABA) receptors and have a similar inhibitory role, are influenced by proinflammatory cytokines [109].

## Some key pathophysiological roles of TNF and IL-1 inside the brain

As we have recently reviewed [110], high brain TNF levels increase harmful cerebral glutamate concentrations through enhancing both glutaminase activity and glutamate re-entry proteins, but evidence implies that IL-1 does not [111]. Clearly, these brain functions are susceptible to TNF and/or IL-1 being above their homeostatic range during a cytokine storm, and consequent change can be expected in the subtleties as well as the gross consequences of excitotoxicity in circumstances when the proinflammatory cytokine load is high and prolonged. Neurogenic pain [112] and insulin resistance in AD brains [113] are examples.

Much relevant material on TNF and the brain that space considerations preclude here is contained in our 2010 review [114]. This text developed from our view that the riddle of malarial encephalopathy (cerebral malaria, (CM)) could be understood only by getting engrossed in understanding how TNF influences brain function in other states, such as AD [115]. We felt that in both of these conditions the historical histological hallmarks – sequestered parasitized erythrocytes in CM, and amyloid (Aβ)plaques in AD – had somehow been allowed, in the absence of other contenders, to evolve into primary mechanisms that researchers were comfortable with, conceivably because they could point them out under a microscope. Increasingly, each hallmark has fallen from the spotlight because of developments in the cytokine storm literature. Importantly, CM has been reported recently to respond dramatically after treating affected mice with the glutamine analog 6-diazo-5-oxo-L-norleucine (DON) [116], lowering the brain glutamate and thus excitotoxicity, that excess brain TNF generates [110].

Likewise, developments in cytokine biology have meant that Aβ, for decades almost universally argued to be central to understanding AD, has lost momentum as the key to this disease as well as its proposed importance in post-stroke syndromes [117] and traumatic brain injury [118]. Over two decades ago TNF was reported to alter synaptic transmission in hippocampal slices [119]. Five years later [120] it was shown that this earlier observation explained the ability of Aβ, through TNF, to do the same. Other laboratories expanded the roles of TNF in this context [103]. The capacity of Aβ to act as a ligand for CD14 and TLR2 indicates that these findings with Aβ [120] are consistent with basic immunology, since occupancy of CD14 and TLRs is how the usual bacterial and protozoal-origin inducers of TNF operate [121]. Key support for this concept has been provided by the recent demonstration that the release of pro-inflammatory cytokines from astrocytes is necessary for either Aβ to be neurotoxic or tau phosphorylation to be initiated [122]. Importantly, a recent large epidemiological study, in which administering regular subcutaneous etanercept, a specific anti-TNF biological agent in common clinical use, over an extended period in treatment of rheumatoid arthritis (RA), was reported to reduce incidence of AD [123] in these patients. This further greatly enhances the likelihood of TNF, and correspondingly decreases the likelihood of Aβ, being the key to AD pathogenesis. These and related arguments are discussed in our reviews [42, 52, 110, 124–128].

More recently a technically impressive study [129] has identified changes in the dynamics of re-uptake proteins, and thus of glutamate, in the extracellular microenvironment near Aβ plaques in the brain of a high Aβ mouse model of AD. Unfortunately, presenting these data as evidence for a direct functional link between Aβ and cognitive impairment and neuronal loss through excitotoxicity, as these authors do, ignores the literature on the essential intermediary role of TNF in these observations [120, 130]. The outcome is actually a strong case for AD arising through a chronic cerebral cytokine storm, since TNF, as well as being induced by many TLR agonists, including Aβ, is well known [110] to influence glutamate dynamics in the way described above [129].

## Clinical usefulness of this knowledge

The obvious inference from the literature reviewed here is that patients suffering from cytokine storms should be treated by neutralize the offending cytokines, mainly TNF. It was, however, established quite early in mice [131] and baboons [132], when knowledge of post-TNF pathways was in its infancy, that specific anti-TNF neutralizing agents have to be administered 1–2 h before TNF induction, while animals are still perfectly healthy, in order to save them. An example of a useful application of this principle has been the successful treatment of the life-threatening Jarisch-Herxheimer reaction that can be induced by the PAMPs released from damaged and dead *Borrelia duttonii*, the cause of African East Coast Relapsing Fever, as a consequence of treating patients with penicillin [133]. Treatment consisted of pre-penicillin exposure to a polyclonal Fab antibody fragments against TNF-alpha (anti-TNF-alpha Fab). In contrast, when someone is acutely ill from sepsis the TNF that made them ill has largely come and gone, having set in train many harmful pathways. This is presumably what prevents specific anti-TNF neutralizing agents from being clinically useful in patients who are acutely ill from sepsis [134]. There is also the practical consideration of anti-TNF agents reducing the efficacy of innate immunity in acute infections, albeit first recognized in treatment of RA patients harboring chronic infections such as tuberculosis, in which innate immunity is an important component [135].

Chronic inflammation is quite another story. Some 5 years ago Karl Nathan reviewed the therapeutic challenge represented by resolving compared to non-resolving inflammatory diseases [136]. Specific anti-TNF biologicals have made their mark in a number of these non-resolving inflammatory states, and their influence is set to expand. They are well-established in the treatment of RA, psoriasis and Crohn’s disease, three non-resolving conditions affecting different anatomical sites in the periphery. Although for historical reasons these three diseases are the province of three different medical specialties, this has not prevented a common approach to treatment. Moreover, treating the non-resolving inflammatory states that constitute the neurodegenerative diseases by this same approach has, for some time now, awakened great interest in some quarters. Unfortunately, this enthusiasm for treating the brain for excess TNF is, to date, largely restricted to neuroscientists and medical specialists with prior anti-TNF experience in their field [123, 137–139]. It has yet, it seems, to extend to neurologists [128], conceivably in part for commercial-in-confidence reasons [140]. Nevertheless, the value for patients in bridging this knowledge gap has been compelling for some years, particularly with recent awareness that brain TNF levels are a main cause of variation in synaptic activity of glutamate, which, across the neurodegenerative diseases, is manifested when in excess as excitotoxicity [110]. As discussed [110], this approach gives an important new level of understanding on the use of not only anti-TNF biologicals, but also nilotinab, 6-diazo-5-oxo-norleucine (DON), 3,6 dithio-thalidomides, ceftriaxone, riluzole, and cannabidiol (CBD) in neurodegenerative states. It also explains [141] the capacity of certain stem cells release to improve post-stroke disabilities through creating an anti-inflammatory milieu by generating large amounts of fibroblast growth factor rather than by replacing dead cells [142].

## Relative importance of TNF and IL-1 as pharmaceutical targets

A useful starting point to discussing this topic is the 1986 report [143] that TNF induces production of IL-1. This is consistent with later studies surrounding the first demonstration of clinical success of infliximab [144], the original specific anti-TNF biological agent, being reported to reduce IL-1 as well as TNF levels [145–147]. It also reduced IL-6 [146, 148], and IL-8 [146]. During these studies this group appears to have been the first to described the concept of a cytokine cascade [146, 148].

Nevertheless, while this literature provides an excellent rationale for the therapeutic use of specific anti-TNF biological agents, there may be an opportunity, particularly regarding IL-1, that is still relatively neglected. There is ample evidence that in certain circumstances IL-1, induced by TNF [143], can itself induce TNF, and the shared and unshared functions of these two cytokines have been discussed at length [149]. These include the membrane-associate IL1 (IL-1α) [150], as well as circulating IL-1, or IL-1β [151, 152]. In addition, TNF and IL-1 can synergize [153–155], for example in causing illness and pathology [156, 157]. Thus, although neutralizing excess TNF has become the dominant pharmacological approach in this field, excess IL-1 is receiving, and certainly warrants, further attention as a target molecule.

TNF and IL-1 received roughly equal prominence in the earlier literature, but neutralizing the effects of TNF has largely dominated the therapeutics literature. In part this may be because specific anti-TNF biologicals have been freely available laboratory and clinical tools, since the mid 1980s and mid 1990s respectively. Two specific anti-IL-1β biologicals, canakinumab [158] and another termed P2D7KK [159], as well as a recombinant form of a naturally occurring antagonist to IL-1 receptor, Anakinra [160, 161], are available. We feel it can fairly be said to date, however, that these agents have proved to be less potent in treating inflammatory disease than are the specific ant-TNF biological agents. In 2013 Dinarello [162] extensively reviewed why this was apparently so. Nevertheless, various applications are still vigorously explored. For example one group has been capitalizing on their finding that IL-1 receptor antagonist (IL-1ra) is an endogenous neuroprotectant that is increased rapidly in a rat model of focal ischemia. This has led them to enhance this antagonist by supplementing its in vivo levels with the recombinant form, Anakinra [163]. They have since reported that, despite a large molecular weight that would preclude its passage through the normal BBB after subcutaneous injection in the rat, Anakinra entered the CSF for a therapeutic window of up to 24 h after the stroke event, and prevented subsequent damage by a reported 33% [164]. This delivery method was also successful after a range of intravenous injection protocols in patients, although clinical outcomes were not measured [165]. An initial controlled trial produced no harm, and was considered too underpowered to achieve positive outcomes [166]. The most likely target for this treatment has been considered to be to increase cerebral blood flow. As noted previously, this group had earlier reported that IL-1ra did not influence glutamate release into synaptosomes [111].

In conclusion, we note that these recent implications of cytokine storms for understanding encephalopathies usefully allow disease pathogenesis to be appreciated as a single entity through bridging the gap between TNF in systemic disease and the brain, as well as encompassing infectious and non-infectious disease on both sides of the blood-brain barrier. This promises new therapeutic perspectives for important cerebral disorders, particularly chronic neurodegenerative states, as exemplified by new data linking cerebral TNF and extra-cellular glutamate referred to earlier in this text. As we have summarized [110], cerebral TNF and consequentially extracellular cerebral glutamate are both chronically increased in Alzheimer’s disease, post-stroke syndromes, traumatic brain injury, and Parkinson’s disease, Huntingdon’s disease, amylotropic lateral sclerosis, septic encephalopathy, poor post-operative cognition, poor post-irradiation cognition, HIV dementia, cerebral malaria and viral encephalitides. Cerebral palsy also fits this pattern [167, 168]. As noted [110], this relationship between TNF and glutamate is consistent with excitotoxicity and synaptic shutdown being major consequences of chronic cerebral cytokine storms, albeit with different anatomical locations, initiators and kinetics. We suggest that the physiological roles of TNF in the brain are sufficiently diverse to generate, when homeostasis of this cytokine and therefore glutamate are lost, most of the overlapping syndromes encompassed by the above array of disease states. Logical therapeutic approaches include specific anti-TNF or anti-IL-1 agents that enter the CSF, whether through their route of administration [139] or size [110]. With these advances, our understanding of cytokine storms is much closer to coming of age.
